# Selective Stimulation of Penumbral Cones Reveals Perception in the Shadow of Retinal Blood Vessels

**DOI:** 10.1371/journal.pone.0124328

**Published:** 2015-04-21

**Authors:** Manuel Spitschan, Geoffrey K. Aguirre, David H. Brainard

**Affiliations:** 1 Department of Psychology, University of Pennsylvania, Philadelphia, Pennsylvania, United States of America; 2 Department of Neurology, University of Pennsylvania, Philadelphia, Pennsylvania, United States of America; University College London, UNITED KINGDOM

## Abstract

In 1819, Johann Purkinje described how a moving light source that displaces the shadow of the retinal blood vessels to adjacent cones can produce the entopic percept of a branching tree. Here, we describe a novel method for producing a similar percept. We used a device that mixes 56 narrowband primaries under computer control, in conjunction with the method of silent substitution, to present observers with a spectral modulation that selectively targeted penumbral cones in the shadow of the retinal blood vessels. Such a modulation elicits a clear Purkinje-tree percept. We show that the percept is specific to penumbral L and M cone stimulation and is not produced by selective penumbral S cone stimulation. The Purkinje-tree percept was strongest at 16 Hz and fell off at lower (8 Hz) and higher (32 Hz) temporal frequencies. Selective stimulation of open-field cones that are not in shadow, with penumbral cones silenced, also produced the percept, but it was not seen when penumbral and open-field cones were modulated together. This indicates the need for spatial contrast between penumbral and open-field cones to create the Purkinje-tree percept. Our observation provides a new means for studying the response of retinally stabilized images and demonstrates that penumbral cones can support spatial vision. Further, the result illustrates a way in which silent substitution techniques can fail to be silent. We show that inadvertent penumbral cone stimulation can accompany melanopsin-directed modulations that are designed only to silence open-field cones. This in turn can result in visual responses that might be mistaken as melanopsin-driven.

## Introduction

**Fig 1:**
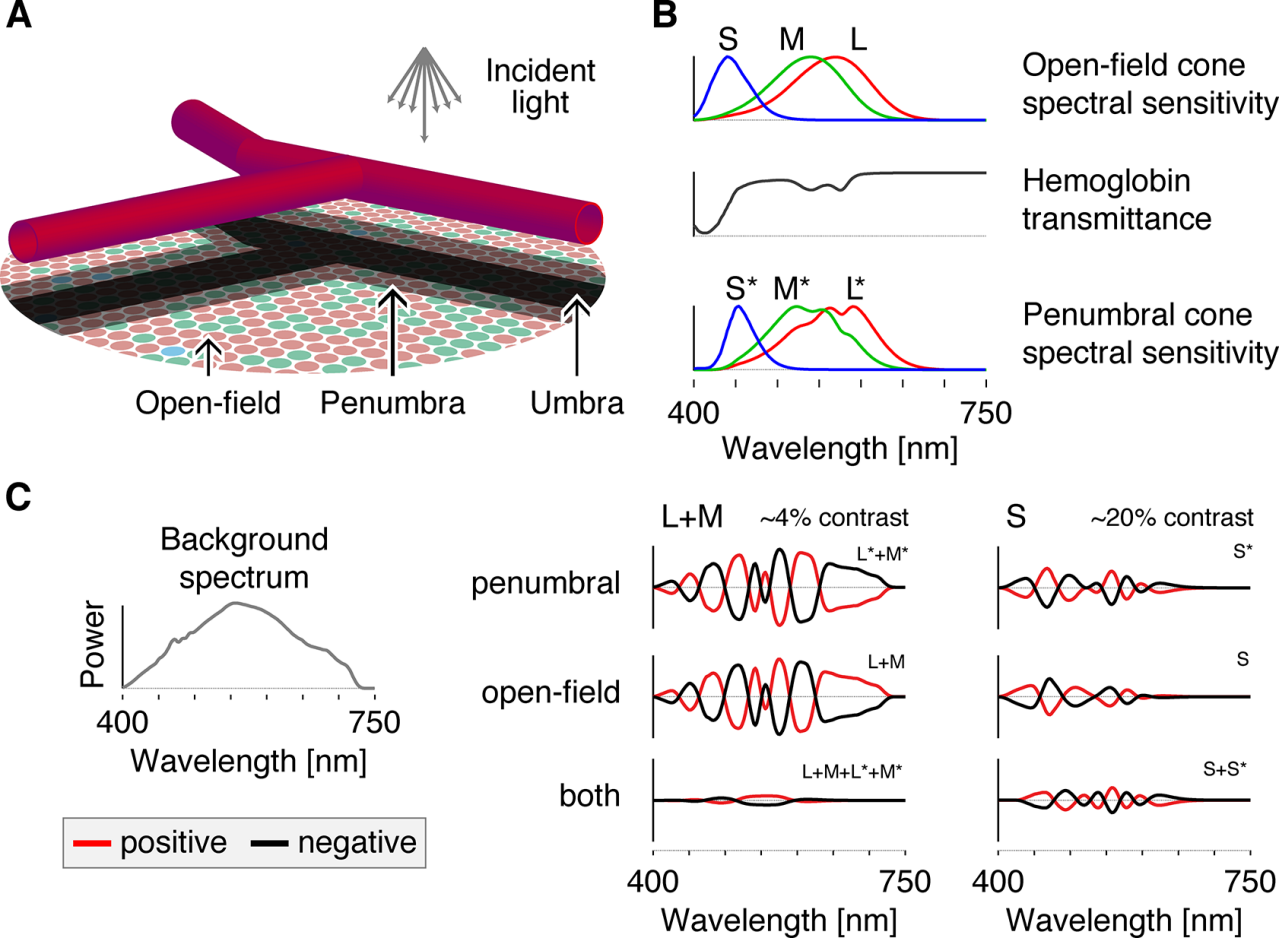
Spectral sensitivities and apparatus. A: Schematic diagram of the retina showing the shadows cast by the retinal blood vessels lying in front of the photoreceptive layer of the retina. B: The spectral sensitivities of the open-field cones (*upper panel*) are filtered by the hemoglobin transmittance spectrum (*middle panel*), resulting in wavelength-specific changes of the cone spectral sensitivities (*lower panel*). C: All modulations are carried out around a rod-saturating background whose spectrum is shown at the left. On the right are plotted the spectral modulations that target each of the indicated cone class(es), with the targeted class(es) indicated at the upper right of each individual plot. The amplitudes of these modulations are varied sinusoidally in time between the plotted positive (red) and negative (black) modulations and are then added to the background spectrum to produce the stimuli seen by the observer.

A fine network of retinal vessels supplies the inner retina with blood [1–3], with decreasing vessel diameter towards the fovea. This network lies in front of the photoreceptive layer of the retina, thus casting shadows onto a set of cone photoreceptors. As the blood vessels are thin, most of the retinal cone mosaic is in the open light field, receiving unobstructed, incident light (Fig 1A). Cones positioned directly under the larger blood vessels lie in deep shadow and are termed *umbral cones*. Between these two regions lies the *penumbra*, in which cones experience partial shadow [4]. As the shadow of the vasculature is stabilized on the retina it is not perceived under normal viewing conditions [5].

**Fig 2:**
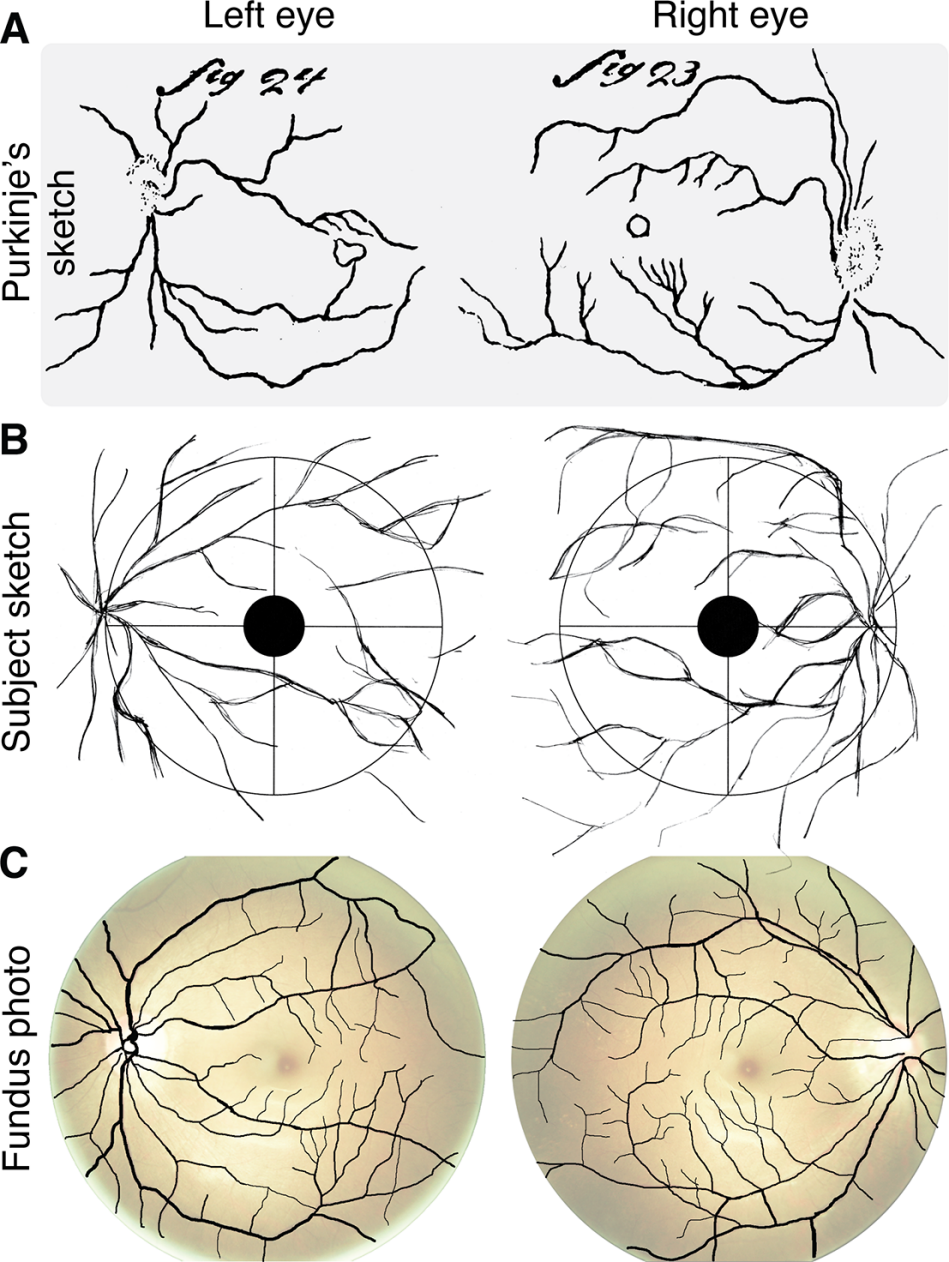
Purkinje-tree percepts. A: Sketch of entoptic visualization of retinal blood vessels from Purkinje ([7]; Figs 23 and 24). B: Sketch produced by a naïve observer in our study while viewing penumbral cone directed flicker at 16 Hz. C: Fundus photographs with overlaid extracted retinal blood vessels (see detailed methods in Appendix). The contrast and brightness of the fundus photographs were adjusted, and then made transparent for visualization purposes (see Fig 5 for raw fundus photographs). The fundus photos were obtained after the observer produced her sketches.

In 1819, the Bohemian physiologist Johann Evangelist Purkinje (Czech spelling *Jan Evangelista Purkyně*) found that moving a candle across the visual field allows an observer to view their own retinal blood vessels. This method displaces the shadow of the blood vessels on the retina, breaking stabilization and producing the entopic percept that we now refer to as the Purkinje tree ([6,7]; see Fig 2A). Visualization of the vasculature using kinetic techniques to move the shadow of the blood vessels has a rich history in vision science [8]. For example, Müller [9, 10] used it to deduce the location of the photoreceptive layer in the retina and more recently it has found a variety of clinical applications [11–34].

Here we demonstrate a novel method for visualizing the Purkinje tree, one that does not involve stimulus motion. The three classes of cones (L, M, and S) differ in their spectral sensitivity (Fig 1B). Light passing through hemoglobin is spectrally filtered. As a consequence, the cones that lie within the shadow of blood vessels have an altered spectral sensitivity relative to their open-field counterparts. The differential spectral sensitivity of open-field and penumbral cones allows these two populations to be selectively targeted using the method of silent substitution [35], with properly tailored spectral modulations. We report that selective stimulation of the penumbral L and M, but not S cones, elicits a clear percept of the retinal blood vessels.

## Results

Using a digital spectral light modulator that produces a mixture of 56 narrowband primaries under computer control, we constructed sets of spatially-uniform spectral modulations that *a*) selectively stimulate penumbral cones (denoted as L*, M* and S*) while silencing open-field cones (denoted as L, M and S), *b*) selectively stimulate open-field cones while silencing the penumbral cones, and *c*) stimulate open-field and penumbral variants conjointly. Each set includes one modulation that targets L and M cones, and a second modulation that targets S cones (Fig 1C; Appendix). Approximately 3–5% contrast was available within the gamut of our device for selective and differential stimulation of the penumbral and open-field L and M cones. That is, to produce more than this amount of contrast we would have to produce power less than zero at some wavelengths and/or power that exceeded that maximum available from our device at others. Approximately 20% contrast was available for the S cone variants. All modulations had zero predicted contrast on melanopsin. Modulations were presented around a high-photopic background (~2000 cd/m^2^, Fig 1C) to saturate the rods. Table 1 provides the specific contrasts and background levels used for each modulation in each experiment. How we determined contrast is described in the Appendix (see Eq 2–4).

**Table 1:** Modulations and contrast values.

		Observer			Chromaticity		Pupil diameter [mm]		Target contrast [%] (Expected absolute contrast splatter [%])							
Direction	Task	Age [yrs]	ID	Mean luminance [cd/m^2^]	*x*	*y*	Assumed	Expected	L	M	S	L*	M*	S*	Mel	Rods
L*+M*	Psychphysical rating task (n = 3) Main data	27	MS	1950	0.38	0.43	3	3.22	0.00 (0.12)	0.00 (0.10)	0.00 (0.32)	**4.24**	**2.64**	0.00 (0.67)	0.00 (0.39)	*2*.*53 (2*.*52)*
		44	GKA					3.06	0.00 (0.11)	0.00 (0.10)	0.00 (0.34)	**4.18**	**2.58**	0.00 (0.69)	0.00 (0.41)	*2*.*66 (2*.*65)*
		54	DHB					2.97	0.00 (0.11)	0.00 (0.11)	0.00 (0.37)	**4.15**	**2.54**	0.00 (0.79)	0.00 (0.44)	*2*.*75 (2*.*75)*
L+M		27	MS					3.22	**4.06**	**2.87**	0.00 (0.31)	0.00 (0.05)	0.00 (0.08)	0.00 (0.65)	0.00 (0.30)	*-1*.*30 (1*.*28)*
		44	GKA					3.06	**3.89**	**2.95**	0.00 (0.32)	0.00 (0.01)	0.00 (0.06)	0.00 (0.66)	0.00 (0.28)	*-0*.*99 (0*.*98)*
		54	DHB					2.97	**3.71**	**3.07**	0.00 (0.34)	0.00 (0.04)	0.00 (0.05)	0.00 (0.73)	0.00 (0.26)	*-0*.*49 (0*.*48)*
L+M+L*+M*		27	MS					3.22	**5.00**	**5.00**	0.00 (0.03)	**4.44**	**4.57**	0.00 (0.05)	0.00 (0.13)	*1*.*89 (1*.*90)*
		44	GKA					3.06	**5.00**	**5.00**	0.00 (0.03)	**4.44**	**4.57**	0.00 (0.05)	0.00 (0.14)	*1*.*91 (1*.*91)*
		54	DHB					2.97	**5.00**	**5.00**	0.00 (0.03)	**4.45**	**4.57**	0.00 (0.06)	0.00 (0.14)	*1*.*91 (1*.*93)*
S*		27	MS					3.22	0.00 (0.04)	0.00 (0.03)	0.00 (0.89)	0.00 (0.05)	0.00 (0.03)	**20.00**	0.00 (0.16)	*-0*.*43 (0*.*43)*
		44	GKA					3.06	0.00 (0.05)	0.00 (0.04)	0.00 (1.06)	0.00 (0.06)	0.00 (0.04)	**20.00**	0.00 (0.21)	*-0*.*68 (0*.*68)*
		54	DHB					2.97	0.00 (0.06)	0.00 (0.06)	0.00 (1.34)	0.00 (0.07)	0.00 (0.05)	**20.00**	0.00 (0.26)	*-0*.*88 (0*.*88)*
S		27	MS					3.22	0.00 (0.02)	0.00 (0.02)	**20.00**	0.00 (0.01)	0.00 (0.01)	0.00 (0.69)	0.00 (0.08)	*0*.*47 (0*.*46)*
		44	GKA					3.06	0.00 (0.04)	0.00 (0.04)	**20.00**	0.00 (0.04)	0.00 (0.03)	0.00 (0.91)	0.00 (0.17)	*1*.*06 (1*.*06)*
		54	DHB					2.97	0.00 (0.11)	0.00 (0.08)	**19.81**	0.00 (0.12)	0.00 (0.07)	0.00 (1.23)	0.00 (0.33)	*2*.*18 (2*.*18)*
S+S*		27	MS					3.22	0.00 (0.04)	0.00 (0.02)	**20.00**	0.00 (0.05)	0.00 (0.03)	**20.00**	0.00 (0.16)	*-0*.*12 (0*.*12)*
		44	GKA					3.06	0.00 (0.04)	0.00 (0.02)	**20.00**	0.00 (0.05)	0.00 (0.03)	**20.00**	0.00 (0.17)	*-0*.*17 (0*.*17)*
		54	DHB					2.97	0.00 (0.04)	0.00 (0.02)	**20.00**	0.00 (0.05)	0.00 (0.03)	**20.00**	0.00 (0.20)	*-0*.*20 (0*.*21)*
Melanopsin A	Psychphysical rating task (n = 3) Supplementarydata	27	MS					3.22	0.00 (0.20)	0.00 (0.20)	0.00 (0.66)	*2*.*93* (2.94)	*2*.*43* (2.42)	*10*.*06* (10.10)	**20.00**	*12*.*78 (12*.*76)*
		44	GKA					3.06	0.00 (0.20)	0.00 (0.21)	0.00 (0.68)	*2*.*85* (2.86)	*2*.*35* (2.36)	*9*.*03* (9.03)	**20.00**	*12*.*75 (12*.*75)*
		54	DHB					2.97	0.00 (0.20)	0.00 (0.25)	0.00 (0.79)	*2*.*81* (2.83)	*2*.*30* (2.27)	*8*.*49* (8.59)	**20.00**	*12*.*74 (12*.*68)*
Melanopsin B		27	MS					3.22	0.00 (0.21)	0.00 (0.18)	0.00 (0.44)	0.00 (0.24)	0.00 (0.21)	0.00 (0.80)	**20.00**	*11*.*86 (11*.*85)*
		44	GKA					3.06	0.00 (0.21)	0.00 (0.19)	0.00 (0.45)	0.00 (0.23)	0.00 (0.22)	0.00 (0.79)	**20.00**	*11*.*80 (11*.*80)*
		54	DHB					2.97	0.00 (0.21)	0.00 (0.23)	0.00 (0.49)	0.00 (0.23)	0.00 (0.26)	0.00 (0.88)	**20.00**	*11*.*77 (11*.*72)*
L*+M*+*Melanopsin*	Sketch	29	HW	2200	0.38	0.43	4.7	3.16	0.00 (0.60)	0.00 (0.97)	0.00 (0.78)	**7.73**	**5.58**	0.00 (2.67)	*41*.*35* (41.05)	*27*.*35 (28*.*05)*

The table provides detailed information on the photoreceptor contrast expected for each of our stimulus modulations. Expected absolute contrast splatter was calculated according to the method outlined in the appendix. L*+M*, penumbral L and M cone modulation; L+M, open-field L and M cone modulation; L+M+L*+M*, modulation visible to both open-field and penumbral L and M cones; S*, penumbral S cone modulation; S, open-field S cone modulation; S+S*, modulation visible to both open-field and penumbral S cones. Individual observer ratings are shown to the right; Melanopsin A, melanopsin-directed modulation that did not silence penumbral cones; Melanopsin B, melanopsin-directed modulation with penumbral cones silenced; L*+M*+*Melanopsin*, penumbral L and M cone modulation ignoring melanopsin. Bold contrast values indicate the targeted photoreceptors. Italic contrast values indicate contrast not controlled for. Expected pupil diameter calculated using the unified formula for light-adapted pupil size from Watson and Yellott [36] assuming a 27.5° field and monocular illumination. This choice affects calculated retinal illuminance and thus the estimated fraction of cone pigment bleached used to construct cone spectral sensitivities. It would have been better to use a 3 mm pupil for observer HW, but the effect of the larger assumed pupil size is small (see Appendix).

The output of the spectral light modulator was imaged onto a diffuser and viewed by observers through a custom eyepiece. The resulting stimulus configuration in all experiments was a spatially uniform annulus with a 27.5° outer diameter and with the central 5° obscured. Thin grid lines were etched into the diffuser to aid accommodation and fixation.

In informal observations, the three authors of this paper observed that when the penumbral L and M cone modulation was flickered at 8 or 16 Hz, a clear percept of the branching retinal blood vessels was produced within the stimulated portion of the visual field. This percept emerged at flicker onset, faded considerably over about a second, and could be restored if the flicker was halted and restarted. We do not have an explanation for the fading. Crucially, when viewing the stimulus with the right eye, the vessels appeared to converge to a point on the right side of the visual field, and vice versa when viewed with the left eye. This mirror symmetry is consistent with the structure of the image of the blood vessels on the retina.

We asked a naïve observer (female, 29 years old) to draw her percept in response to a penumbral-cone directed spectral modulation. She viewed a 16 Hz square-wave modulation of penumbral L and M cones monocularly with each eye in turn (the modulation viewed by this observer differed from those studied further below in that it did not silence melanopsin and consequently allowed a larger penumbral cone contrast; see Appendix and Table 1). The observer viewed the modulation and drew her entopic percept on a diagram that indicated both the obscured central 5° and the central grid lines superimposed on the uniform stimulus field (as shown in Fig 2B; the circle shown corresponds to the size of the 27.5° diameter field). The observer freely switched between the drawing and viewing the stimulus. The sketches produced by this observer are notable for their general resemblance to a Purkinje tree and may be compared with her actual vasculature as obtained from ocular fundus photographs of her two eyes (Fig 2C). The sketches capture the gross features of the larger blood vessels seen in the photos, with the larger vessels in the sketch emanating from the optic disk in both eyes and with smaller vessels shown branching from the larger ones. The implied location of the optic disks in the sketches (just inside the 13.75° eccentricity indicated by the circle) is consistent with their known anatomical positions in the retina [37]. A more detailed examination reveals that the correspondence between the sketches and photos is not exact; this may be a result of limits in the sketching ability of the observer. We also attribute to sketching imprecision the fact that the sketches extend slightly beyond the indicated outer diameter of the stimulus field; the Purkinje tree percepts were always confined to the part of the visual field that was stimulated.

Consistent with our own percepts, the sketched Purkinje tree corresponds only to the larger retinal blood vessels, similar in spatial structure to those observed when a moving penlight is shown through the side of a closed eyelid. Finer vasculature extending in towards the fovea, which may be observed using other kinetic methods, was not visible using our method.

We conducted a rating experiment to measure three properties regarding the photoreceptor contributions to and temporal characteristics of the Purkinje-tree percept. First, we tested whether penumbral cone stimulation is necessary to produce the Purkinje-tree percept, or whether differential contrast between penumbral and open-field cones is both necessary and sufficient. Second, we considered that spatial contrast produced by the uniform spectral flicker is perfectly stabilized on the retinal surface, as the blood vessels move with the photoreceptors. Thus we predicted that the temporal dependence of the Purkinje-tree percept should have a bandpass shape similar to that found in measurements of temporal contrast sensitivity for retinally stabilized images [38,39]. Finally, we expected the Purkinje-tree percept to be more robust for penumbral L and M cone flicker as compared to penumbral S cone flicker, given the reduced spatial resolution of the S cone mosaic.

**Fig 3:**
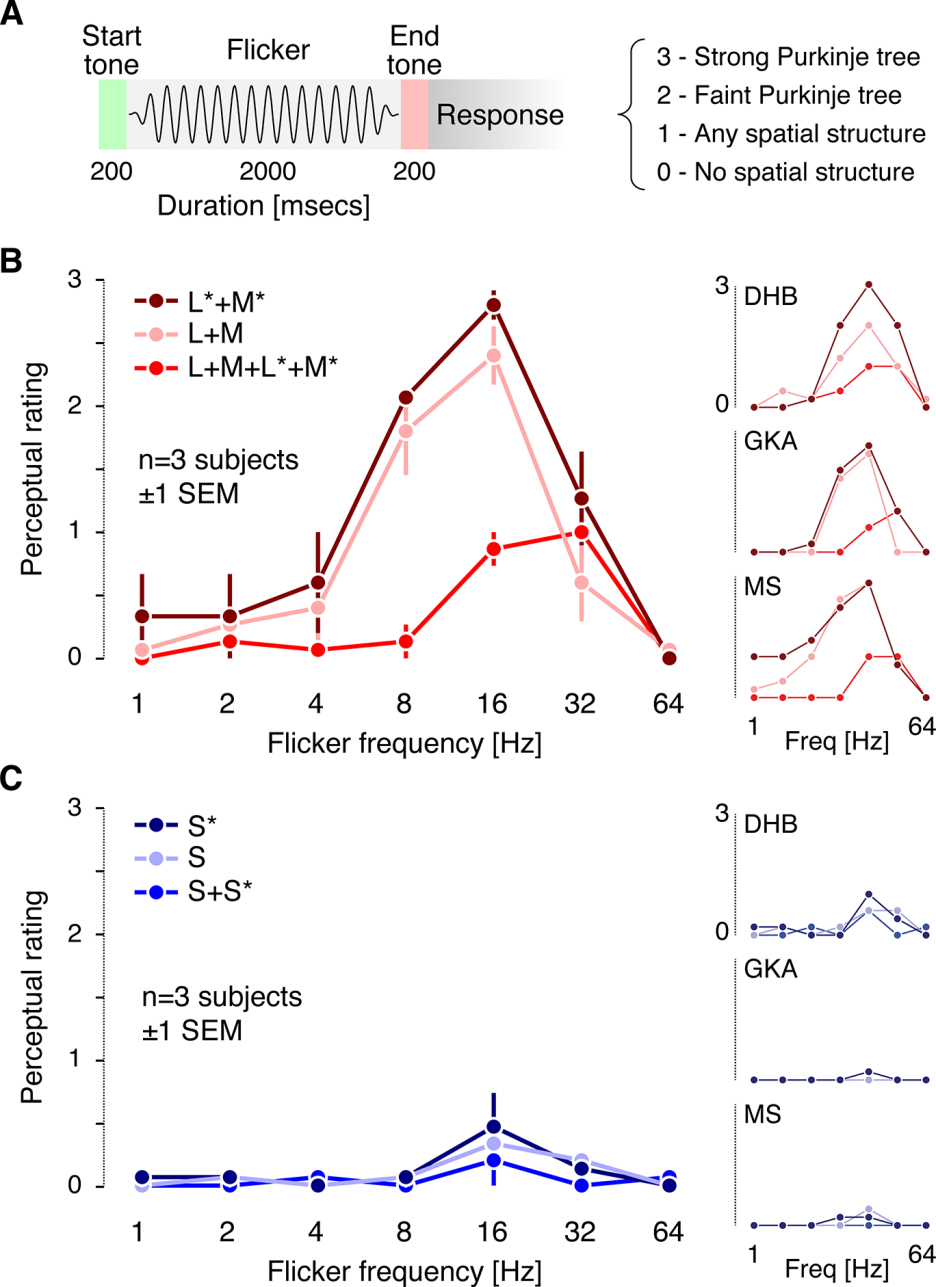
Psychophysical rating results. A: Time course of a single trial of the rating experiment and summary of the perceptual rating scale (see main text for more detailed description). B: Average ratings across the three observers for the L and M cone directed modulations. L*+M*, penumbral L and M cone modulation; L+M, open-field L and M cone modulation; L+M+L*+M*, modulation visible to both open-field and penumbral L and M cones. Individual observer ratings are shown to the right. C: Average ratings across the three observers for the S cone directed modulations. S*, penumbral S cone modulation; S, open-field S cone modulation; S+S*, modulation visible to both open-field and penumbral S cones. Individual observer ratings are shown to the right.

The three authors viewed 2-second trials consisting of sinusoidal flicker modulations, with 250 ms cosine windowing at the beginning and end of each trial. Tones demarcated the start and end of each trial (Fig 3A). The six spectral modulation directions depicted in Fig 1C were shown. Each combination of modulation direction and frequency was presented five times, with trial order randomized. The observer was blind to the particular modulation direction and frequency presented on each trial. On every trial, the observer was asked to rate his percept using a 0–3 scale (0 = little or no spatial structure visible in the flicker; 1 = some spatial structure visible, but structure did not resemble the Purkinje-tree percept; 2 = faint or partial Purkinje-tree percept visible; 3 = strong Purkinje-tree percept). A rating of 3 corresponded to the strongest percepts we had observed in our apparatus. These were very salient, as in the sketch shown in Fig 2. On 21 out of 231 trials, no modulation at all was shown (blank trials), to provide a control for false positives.

Both the selective penumbral L and M cone and the open-field L and M cone modulations elicited a clear Purkinje-tree percept at 16 Hz in all three observers (Fig 3B), with the strength of the percept falling off at lower and higher temporal frequencies. The control L and M cone modulation, which stimulated both open-field and penumbral L and M cones with similar contrast (much as would occur during natural viewing), did not elicit a Purkinje-tree percept. This confirms that time-varying differential contrast between open-field and penumbral cones is required to elicit a Purkinje-tree percept, and that this percept can be elicited by selective modulation of either penumbral or open-field cones.

The control L and M cone modulation did elicit faint percepts of spatial structure at temporal frequencies at and above 16 Hz, consistent with previous reports of flicker-induced visual field articulation at high frequencies [7,40–44]. All observers noted that this percept was markedly different from that of the Purkinje tree. Blank trials did not lead to reported spatial structure (MS and GKA, all trials rated as 0; DHB 20 of 21 trials rated as 0, 1 trial rated as 1).

In contrast, selective stimulation of penumbral or open-field S cones did not produce a Purkinje-tree percept for any observer at any temporal frequency, although these modulations could produce some non-specific spatial structure at 16 and 32 Hz (Fig 3C). Given the similarity of the temporal frequency dependence of the S cone ratings to those for L+M+L*+M*, it is possible that the S cone data are driven by residual contrast seen by L and M cones, as the precision of photoreceptor isolation is never perfect (see Appendix).

The trial-by-trial data for the rating experiment as well as the spectra of the modulations used are provided at http://dx.doi.org/10.6084/m9.figshare.1327815. This site also provides the data for the supplemental rating experiment (see below) and the spectra of the modulation presented to our naïve observer.

## Discussion

A variety of methods to visualize one’s retinal vasculature have been described since Purkinje’s original observation. These have included: the use of stimulus motion under special conditions, most notably moving a small spot of light across the pupil [45]; very brief flashes illuminating the retina through the sclera causing a brief shift in the retinal position of the shadow of the vasculature [46]; the motion of corpuscles flowing through the vasculature [14,47–49]; long adaptation to dark, for example after lid closure during sleep [5,50–55]; intense illumination [56,57]; and pressure-induction [10,47,58]. These methods have been employed in a variety of studies that have determined the parameters needed to optimize entopic visualization [12,46,59–63].

Here we demonstrate a novel method for visualizing the Purkinje tree. Differential modulation of the L and M cones inside and outside the partial shadow of the retinal blood vessels produces a strong percept of one’s retinal vasculature, when viewed at 16 Hz. We formalized this observation through a rating experiment, as well as through the sketches of a naïve observer. Our method is conceptually distinct from earlier kinetic techniques for blood vessel visualization: what changes over time in our method is the spectrum of a spatially uniform stimulus. This change in spectrum, rather than spatial motion of the shadow, stimulates penumbral cones differentially from the neighboring open-field cones and leads to the characteristic Purkinje-tree percept. Because our method does not involve spatial modulation of the image impinging on the photoreceptors, it provides a new tool for studying percepts arising from retinally stabilized images. For example, future work could use this technique to investigate in detail the fading of the Purkinje tree percept that we observe for continuously flickering stimuli.

Interestingly, the possibility of a spectral stimulus modulation that selectively drives the penumbral cones was suggested 50 years ago by Cornsweet [64], who wrote:

“[I]t should be possible to provide a visual field in which the only thing that is changing is the stimulation of receptors behind blood vessels. This may be accomplished by showing the observer a field that is alternately lighted with 415 mμ [nm] and then with a mixture of two other wave lengths each of which is absorbed less strongly by blood, but so chosen that their mixture will match the 415 mμ [nm] light in regions not lying behind blood vessels.” (p. 173)

The method we present here may be considered an implementation of Cornsweet’s concept, albeit accomplished by a more complex spectral modulation than he envisioned. Our ability to construct an appropriate modulation is enabled by much more precise estimates of the cone fundamentals and their variation across the retina than were available in the 1960s [65–67].

We found that a temporal frequency of 16 Hz led to the strongest percept of the Purkinje tree, with weaker percepts at 8 and 32 Hz. These frequencies are somewhat higher than those reported by Sharpe [59] for visualization of the Purkinje tree using a kinetic method, and higher than the peak of the temporal contrast sensitivity functions for stabilized retinal images [38,39]. In the former case differences might be expected because our stimulus does not involve any retinal motion, and in the latter because the temporal contrast sensitivity functions will depend on retinal location and the spatial structure of the stimulus as well as possibly the quality of image stabilization. Coppola and Purves [46] concluded from their kinetic-based studies of the Purkinje-tree percept that optimal stimulation frequencies were greater than 10 Hz. Our results are commensurate with this conclusion, although the data on which it is based were obtained for more central visualization of small capillaries rather than for the shadows of larger peripheral vasculature revealed by our method.

In a series of careful anatomical studies, Adams and Horton [4, 68, 69] demonstrated that the photoreceptors under retinal blood vessels have a corresponding area of decreased cortical representation in the squirrel monkey visual cortex, akin to a local form of amblyopia. The altered cortical representation was present even for smaller vessels that produce only penumbral shadow, and the width of the reduced cortical representation was wider than the shadows themselves. We find that the selective stimulation of the penumbral cones results in a visible percept. Thus, the reduced cortical representation identified by Adams and Horton [4, 68, 69] is not so extreme as to render penumbral cones inoperative or to eliminate the possibility of spatial vision mediated by these cones.

Our result has practical implications for the study of visual processes. For example, there is current interest the melanopsin-containing intrinsically photosensitive retinal ganglion cells [70–92]. Studies of melanopsin function in the mouse are facilitated by the use of transgenic photoreceptor knock-out models [75,76,93]. These techniques are obviously not available for studies of human observers. Because of the overlap in spectral sensitivity of the cones and melanopsin, functional isolation of melanopsin in humans has been approached using the method of silent substitution [84,90,94–96]. As typically implemented, modulations that target melanopsin and silence the open-field cones will produce residual stimulation on the penumbral cones. In their recent paper, Horiguchi et al. [82] calculated that such residual stimulation exceeds the cone detection threshold and noted the possibility that the effects of such stimulation might account for some of their psychophysical results, particularly the surprising observation that in the periphery observers could detect nominally cone-silent modulations at 40 Hz. We calculated that the nominally melanopsin-isolating direction in the study of Horiguchi et al. [82] produced 1–2% contrast on penumbral cones. In our recent study of photoreceptor contributions to the pupillary light reflex [94], the primary melanopsin-directed modulation employed also produced residual stimulation of penumbral cones of similar magnitude. Our demonstration here that a small degree of selective penumbral cone contrast produces a clearly visible percept should prompt caution regarding the interpretation of results obtained using silent substitution, particularly under conditions where penumbral-cones might plausibly mediate the measured response of interest. Similar warnings have been issues previously regarding variation of cone spectral sensitivity across the retina as a function of changes in pre-retinal filtering [97,98] and with respect to individual variation in cone spectral sensitivities [82].

It is possible to produce spectral modulations that target melanopsin with about 20% contrast while nominally silencing both open-field and penumbral cones. Indeed, in a supplemental rating experiment, we found that controlling for the stimulation of penumbral cones reduces or removes the Purkinje-tree percept, which is otherwise visible for a melanopsin-directed modulation (see Appendix). While even a small degree of penumbral cone contrast produces a prominent perceptual effect at 16 Hz, we have found that failure to control for this inadvertent stimulation has minimal effect upon measured pupil responses at low temporal frequencies [94]. Consequently, the degree of attention needed to the effect of cones lurking in the vascular shadows will vary, depending upon the response being measured.

## Appendix: Detailed Methods and Supplemental Experiment

### Subjects

The three authors of the study served as observers. All three are male, have corrected visual acuity of 20/20 or better and normal color vision as judged by Ishihara color plate screening [99]. At the time of data collection their ages were: MS 27, GKA 44, DHB 54. The research was conducted in accord with the principles of the Declaration of Helsinki and approved by the University of Pennsylvania Institutional Review Board. Informed written consent was obtained from all observers.

### Apparatus

The apparatus is discussed in detail in Spitschan et al. [94]. We used the method of silent substitution [35,100] in combination with a spectral light modulator (OneLight VISX Spectra Digital Light Engine), which produces light with arbitrary spectral power distributions. The theory of operation of the modulator is as follows: Light from a Xenon arc lamp passes through a slit, is collimated, then passed through a diffraction grating. This separates the light into individual narrowband wavelength components. Each component is then imaged onto a column of a digital light processing (DLP) chip (1,024 columns x 768 rows). Each row on this chip can be turned on or off independently in each column, thus allowing for the selective control over the exitant power in each wavelength band. Rather than addressing the 1,024 columns separately, we treated groups of 16 columns as single primaries, resulting in 56 independent nearly monochromatic primaries (after turning off 80 columns at the short wavelength end of the spectrum, and 48 columns at the long wavelength end of the spectrum, where there was too little light power for us to measure accurately). This grouping of columns provided us with 768 x 16 = 12288 discrete power levels for each primary; the spectral width of the primaries (~16 nm FWHM) was dominated by the spectral bandwidth of the optical system rather than the width of a column on the DLP chip, and there was little spectral shift with output power for our 56 primaries. The DLP chip can modulate rapidly. In the experiments reported here we use it in a mode where we control the emitted spectra at 256 Hz.

The monochromatic primaries leaving the DLP chip were mixed and transmitted through a fiber optic cable (FTIIG16860-40, total length 40 feet; Fiberoptics Technology, Inc.), passed through IR and UV blocking filters, and illuminated a diffuser within a custom-made eye piece. Observers viewed an image of the diffuser through a 25 mm focal length lens, resulting in a 27.5° spatially uniform field with the central 5° blocked by an opaque circular occluder mounted on the diffuser. The diffuser also contained then etched grid lines to facilitate accommodation and fixation by the observer.

The power at each wavelength for each of the 56 primaries was measured through the eyepiece using a spectraradiometer (PR-670 SpectraScan, Photo Research). Each primary was measured at 16 power levels, which allowed us to characterize the nonlinearities between the device primary settings and the exitant light power. We verified that the light emitted from the spectral modulator with all mirrors turned on contained no appreciable power in the UV (200–380 nm) or NIR (780–1020 nm) wavelength ranges using fiber spectrometers (two customized Ocean Optics USB2000+ spectrometers with ILX-511B detector; wavelength ranges 180 nm–875 nm and 340–1025 nm, respectively; 3 m custom Ocean Optics fiber-optic cable). We calculated that the light power in the visible range of the spectrum (380–780 nm) was within light safety standards [101] and provide example code for light safety calculations in the Silent Substitution Toolbox (see below, http://silentsubstitutiontoolbox.org).

### Construction of Spectral Modulations

Because we have available 56 primaries, there are many physically distinct spectral modulations that can be produced by our device and that satisfy a set of specified photoreceptor-silencing constraints. To choose a specific modulation, we have developed general methods that allow us to trade off between several criteria. First, we wanted spectral modulation directions that maximized contrast on targeted photoreceptor classes, within the gamut limits of our stimulation device [102,103]. Second, to the extent possible, we wanted the modulation to vary smoothly with respect to wavelength. This second requirement was imposed based on the intuition that small deviations between the desired modulation and that actually produced by the device will have a smaller effect on nominally-silenced photoreceptors for spectrally smooth modulations than will modulations that vary rapidly as a function of wavelength. Third, we wanted the contrasts produced by the modulations, particularly their ability to silence photoreceptor classes, to be robust with respect to uncertainty in our estimates of the spectral sensitivity of the nominally silenced photoreceptors. To choose modulations that are consistent with these criteria, we implemented a constrained numerical optimization procedure. We have made our software available under an open-source license (Silent Substitution Toolbox; http://silentsubstitutiontoolbox.org). This provides a MATLAB implementation, as well as methods for obtaining estimates of photoreceptor spectral sensitivities (in conjunction with software and data provided in the open-source Psychophysics Toolbox; Brainard [[104], http://psychtoolbox.org]) and methods for computing what we refer to as contrast splatter maps (see Precision of Photoreceptor Isolation section below). Our software takes advantage of the sequential quadratic programming (SQP) algorithm provided in the function *fmincon* in MATLAB’s Optimization Toolbox.

To find desired modulations in the 56-dimensional device primary space, we divide the photoreceptor classes under consideration into three sets. We call these the targeted set, the silenced set, and the ignored set. We search over device primary settings to maximize the contrasts seen by the photoreceptor classes in the targeted set, subject to the constraints i) that the contrasts seen by the photoreceptors in the silenced set are zero and ii) that the device primary settings are within gamut. More specifically, we minimize the quantity

$$f=\sum_{m}{\left(C_{m}-1\right)}^{2}$$ (1)

where *m* indexes the photoreceptor classes in the targeted set and *c*_m_ is the contrast seen by the *m* ^th^ class in the set. The quantity *f* is minimized when the contrasts in the targeted set are as close to 1 as can be obtained subject to the constraints described below. We generally seek spectral modulation directions for which equal positive and negative contrasts around the specified background may be obtained within gamut around the specified background, so that 1 is the maximum obtainable contrast. As a practical manner, our code enforces the device gamut constraints for both the positive and negative modulations during the numerical optimization.

The optimization also enforces a smoothness constraint on the modulations: the maximum absolute difference in spectral power between adjacent sample wavelengths was required to be below a specified criterion. The exact choice of criterion depends on the size of the wavelength sampling step and the intensity of the spectral background, and it can be adjusted to tradeoff between maximal obtainable contrasts for the targeted set and the spectral smoothness of the obtained modulation spectra. This is a choice we make manually for each application of our procedures. For the modulations used in this paper, the wavelength sampling was 380 nm to 780 nm in 2 nm steps, the criterion was 10^−1.5^ watts/[m^2^·sr] relative to a background spectrum with total radiance of about 11.7 watts/[m^2^·sr].

To compute the contrasts for each photoreceptor class, we use device calibration information to compute the predicted spectrum from the device settings. This, together with specification of a background spectrum, allows the computation of contrasts. Typically, we chose the background spectrum *B*(λ) to be that produced by the mixture of all primaries at half their maximum power. Suppose there are *n* classes of photoreceptor under consideration, whose spectral sensitivities *S*_*n*_(λ)are known. We compute the receptor responses of each class to the background *b*_*n*_ as

$$b_{n}=\sum_{n}S_{n}(\lambda )B(\lambda )\delta \lambda$$ (2)

Similarly, for any other spectrum *M*(λ), we compute the receptor responses *m*_*n*_ as

$$m_{n}=\sum_{n}S_{n}(\lambda )M(\lambda )\delta \lambda$$ (3)

This yields the contrast seen by each photoreceptor class between the between *M*(λ) and *B*(λ) as

$$c_{n}=\frac{m_{n}-b_{n}}{b_{n}}$$ (4)

To make the silent-substitution properties of the modulations robust to uncertainty about the spectral sensitivities of the specified photoreceptor classes, we add to the silenced set of photoreceptors not only the nominal versions of the silenced photoreceptor classes but also variants of the nominal versions that represent individual variation. Effectively, the set of silenced photoreceptors provides a basis that spans a larger space of silenced photoreceptor sensitivities. For example, if we are silencing the open-field cones, we have the option of adding to the silenced set variants of the open-field cones computed with higher and lower lens densities than the nominal versions. Whether this is desirable to do again involves a tradeoff that we make manually. The more photoreceptor variants we add to the silenced set, the more robust the modulations but the lower the maximum contrast achievable for the targeted set. We make this tradeoff through examination of the maximum achievable contrasts and of “contrast splatter maps”, which are described below. For the modulations used in this paper, we did not in the event add additional photoreceptor classes to the silenced set, because we wanted to produce as much differential contrast as possible between penumbral and open-field cones. In other work [94], we have found it beneficial to increase robustness in the manner described here.

Sometimes it is desirable not to maximize the contrast for the photoreceptor classes in the maximized set, but rather to produce modulations that have specified target contrasts for each class in this set. For example, for the supplemental experiment reported in this paper we produced modulations that had the same 20% contrast on melanopsin with and without stimulating the penumbral cones. This is achieved in our software by replacing the objective function that seeks to maximize the contrast of the classes in the maximized set with a modified objective function that seeks to bring these contrasts as close as possible to a set of specified target contrasts *t*_*m*_. Specifically, in this case the optimization routine seeks to minimize the sum of squared deviations

$$f=\sum_{m}{\left(C_{m}-t_{m}\right)}^{2}$$ (5)

between the predicted contrasts *c*_*m*_ and the targeted contrasts *t*_*m*_ for the members of the targeted set. In practice, we generally begin by finding the maximum available contrast on classes in the maximized set and then choosing targeted contrasts based on this information.

The contrasts of the photoreceptor classes in the ignored set are disregarded in the optimization. For example, when we compute modulations for use in studies at high photopic light levels, we often place the rod spectral sensitivities in the ignored set. The reader may note that this could also be accomplished simply by not specifying the ignored classes in the set of photoreceptor spectral sensitivities under consideration. We find the code more transparent if we allow for explicit specification of what classes are ignored, as this allows use of a single set of receptor sensitivities across multiple calls to the optimization routine. The calculation of a given desired spectral modulation takes <400 ms in MATLAB on a current (2014) desktop computer.

### Estimates of Photoreceptor Sensitivities

Spectral sensitivities of the open-field L, M and S cones at the cornea were calculated using the CIE 2006 parametric model and incorporate pre-retinal filtering due to lens, ocular media and macular pigment using the age, pupil size and field size dependences of that model [66]. Pre-retinal filtering for all spectral sensitivities was computed using the actual age of each observer and the appropriate field size (27.5°). Although the CIE standard specifies a very small but non-zero amount of macular pigment density outside the central 5°, the contribution of macular pigment filtering to the cone fundamentals for our stimulus configuration is minimal. We adjusted the peak optical density of the cone photopigments depending on the expected proportion of bleached photopigment [105,106] for the retinal illuminance of our rod-saturating background stimulus. For the psychophysical rating task (observers MS, GKA, DHB), we assumed a pupil diameter of 3 mm in computing retinal illuminance, corresponding to the natural light-adapted pupil. For the sketch drawing, we assumed a pupil diameter of 4.7 mm, which is slightly higher than the expected light-adapted pupil size for that light level (Table 1; [36]).

We obtained spectral sensitivities for the penumbral cones by assuming that hemoglobin acts as a pre-retinal filter. We calculated the hemoglobin transmittance spectrum as follows, following the calculations of Horiguchi et al. [82]. We obtained estimates of the molar extinction coefficients *e* of oxyhemoglobin (HbO_2_) and deoxyhemoglobin (Hb) expressed in [cm^−1^/(moles/liter)] [107]. To convert this to the absorptivity, we multiplied by the constant 2.303 and the molar concentration of oxyhemoglobin and deoxyhemoglobin, given by $\frac{\text{150 gHb/liter}}{\text{64,500 gHb/mole}}$ [107], giving the *absorptivity* coefficients $A_{{\text{HbO}}_{\text{2}}}$ and *A*_Hb_, expressed per μm. We assumed an optical path length through the vessels of 11.5 um for penumbral cones, thus obtaining *absorptance* coefficients $a_{{\text{HbO}}_{\text{2}}}=11.5A_{{\text{HbO}}_{\text{2}}}$ and$a_{\text{Hb}}=11.5A_{\text{Hb}}$. This diameter corresponds to the size of venules or smaller arterioles. We combined these to get overall absorptance as follows. We assumed that oxyhemoglobin makes up 95% of the hemoglobin in arteries and 75% of the hemoglobin in veins at room air oxygenation following the oxygen-hemoglobin dissociation curve of adult hemoglobin [108], and took the average between these two to set the fraction of oxygenated hemoglobin at 85%. The overall absorptance was computed as $0.85a_{{\text{HbO}}_{\text{2}}}+0.15a_{\text{Hb}}$ and the transmittance was then obtained as$10^{-(0.85a_{{\text{HbO}}_{\text{2}}}+0.15a_{\text{Hb}})}$.

We constructed the spectral sensitivity of melanopsin along the lines of a recently proposed standard for ‘melanopic’ sensitivity [109] by shifting the Govardovskii nomogram [110] to have its peak spectral sensitivity *λ*_max_ at 480 nm, consistent with previous reports of the peak spectral sensitivity of melanopsin [111–113]. We assumed an optical density of 0.015 [114]. For melanopsin, pre-retinal filtering was incorporated as for the cones, except for filtering due to macular pigment, which was omitted altogether because the melanopsin-containing retinal ganglion cells lie in front of the macular pigment layer.

### Precision of Photoreceptor Isolation

**Fig 4:**
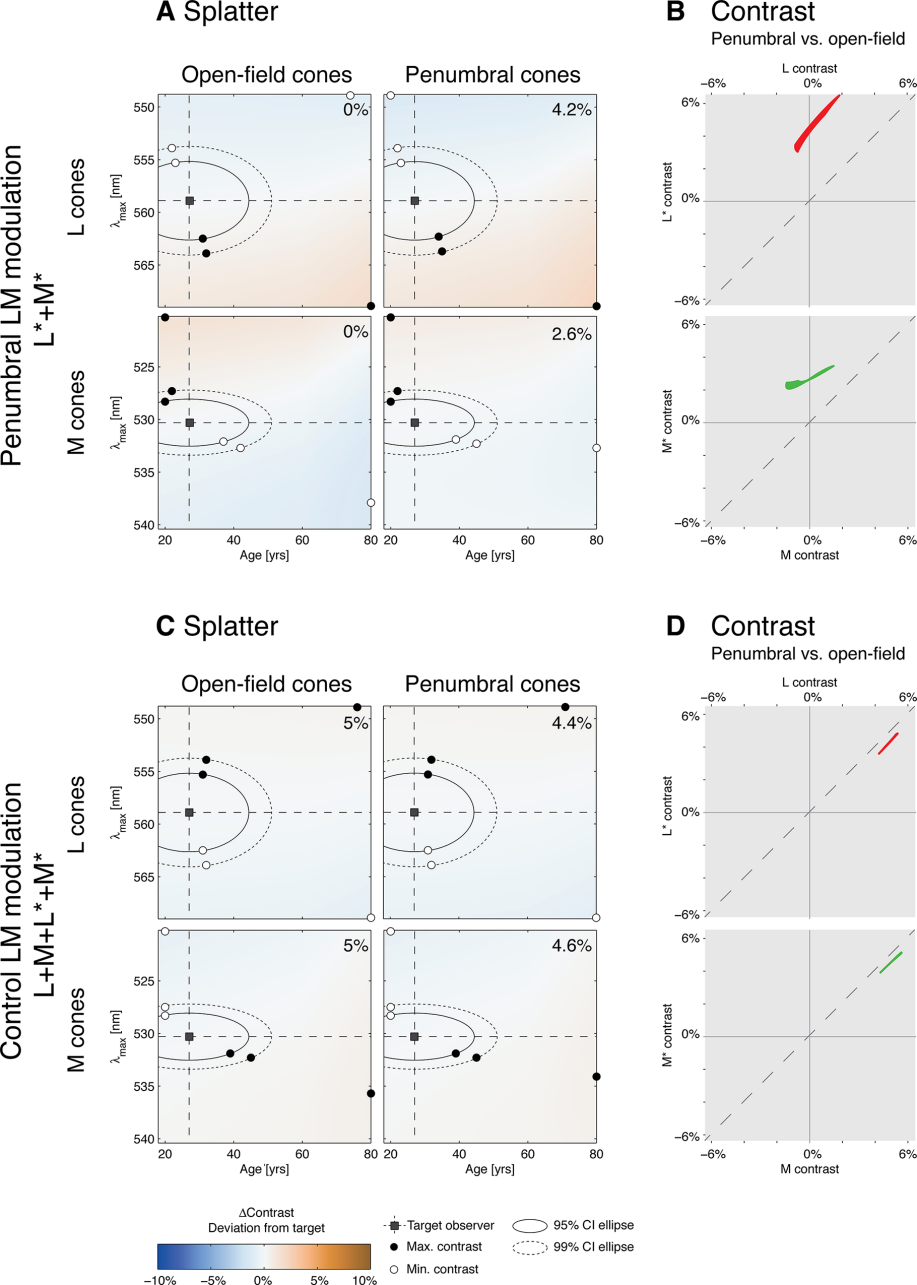
Contrast splatter. A: Contrast splatter calculations for the penumbral L and M cone (L*+M*) modulation (27 year old observer). Separate splatter maps for open-field and penumbral L and M cones are provided. Each point in a splatter map indicates in pseudocolor the contrast that will be seen by a variant of the nominal cone spectral sensitivity, as indicated by its position on the age and *λ*_max_ axes. The color scale is provided at the bottom of the figure, with negative contrast splatter indicating contrast splatter that is 180° out-of-phase with the nominal stimulus modulation. The open square indicates age and *λ*_max_ of the nominal cone spectral sensitivity, while the solid and dashed ellipses indicate the 95% and 99% confidence ellipses for variation around the nominal sensitivity. Open and closed circles on each ellipse show the variant with the minimum and maximum contrast splatter on the ellipse. Open and closed circles on the edges of the map represent the variant with minimum and maximum contrast splatter over the whole range of variants computed. The nominal contrast of the modulation for each cone type is provided in the upper right of each map. B: Comparison of contrast seen by the penumbral vs. open-field L cones across the entire range of photoreceptor variants studied in panel A (top plot) and similarly for the M cones (bottom plot). C: Contrast splatter maps for the modulation that stimulated both penumbral and open-field L and M cones together (27 year old observer). Same format as panel A. D: Same type of comparison as shown in panel B, obtained from the splatter maps shown in panel C.

To estimate the uncertainty of the method of silent substitution given our apparatus, we calculated *contrast splatter*, which is the expected amount of contrast on nominally silenced photoreceptor classes for a given modulation around a given background. This was done by calculating contrast across variants of photoreceptor sensitivity obtained by shifts in the assumed wavelength of photopigment peak absorbance (*λ*_max_) and varying observer lens density using age as the parameter describing lens density according to the CIE formula [66]. Shifts of photopigment absorbance were accomplished by using the Stockman-Sharpe nomogram, which provides a formula that yields the full photopigment spectral absorbance spectrum given a specified wavelength of peak absorbance *λ*_max_ [65]. A calculation was performed for each photoreceptor class in which we varied *λ*_max_ by ±10 nm and let the age parameter vary between 20 and 60 years. In estimating the spectral sensitivities of the photoreceptor variants, we did not recalculate the estimate of fraction of cone photopigment bleached for each variant. Fig 4 shows the results of the splatter calculation. In panel A, for example, the top two pseudocolor plots show the computed contrast splatter map for each variant of the open-field L cones and penumbral L cones, for a 27 year old observer (MS), for the L and M penumbral cone modulation. Computed contrast matches the nominal values (0% for open-field L cones; 4.2% for penumbral L cones) for the targeted *λ*_max_ (558.9 nm) and observer age (27), and deviates from the nominal values for other L cone variants.

Using estimates of the variability of *λ*_max_ and lens density in the population of normal color observers, we then constructed 95% and 99% confidence ellipses for these parameters, based on the assumption that variability is normally and independently distributed for both *λ*_max_ and lens density. We also calculated the expected absolute contrast splatter, which is the expected absolute value of the deviation between obtained and targeted contrast for the silenced photoreceptors, based on the same assumption. We assumed standard deviations of 1.5, 0.9 and 0.8 nm for L, M and S cone *λ*_max_ variation around the nominal values [115]. We extracted the standard deviation of the veridical measurement residuals vs. chronological age from a two-component lens density model [116] and found that the standard deviation of the predicted age parameter of lens density due to individual variation is 7 years. For nominally silent photoreceptor classes, the expected absolute contrast splatter does not exceed 1.23% for any observer, modulation direction, and photopigment absorbance variant (Table 1). Additional contrast splatter may arise because of limitations of stimulus control. We periodically assess this via direct measurement of the spectra produced by our stimulus device, and find that it is of the same order as the contrast splatter we expect from uncertainty in photoreceptor sensitivities.

We considered the possibility that the modulation that nominally drove open-field and penumbral cones together (L+M+L*+M*) in practice elicited substantial differential contrast between the open-field and penumbral cones. To test this, we constructed contrast splatter maps for this modulation (Fig 4, panel C) for the 27 year old observer (MS). For each photoreceptor variant we then plotted the contrast seen by the penumbral L and M cones against the contrast seen by their open-field counterparts (Fig 4, panel D). We find that the contrasts seen by the open-field and penumbral cones are similar to each other across all of the photoreceptor variants, indicating that this key property of the modulation is highly robust with respect to variation in photoreceptor spectral sensitivity. The differential contrast of penumbral cones relative to open-field cones for our penumbral L and M modulation is similarly robust (Fig 4, panel B).

### Fundus Photos

**Fig 5:**
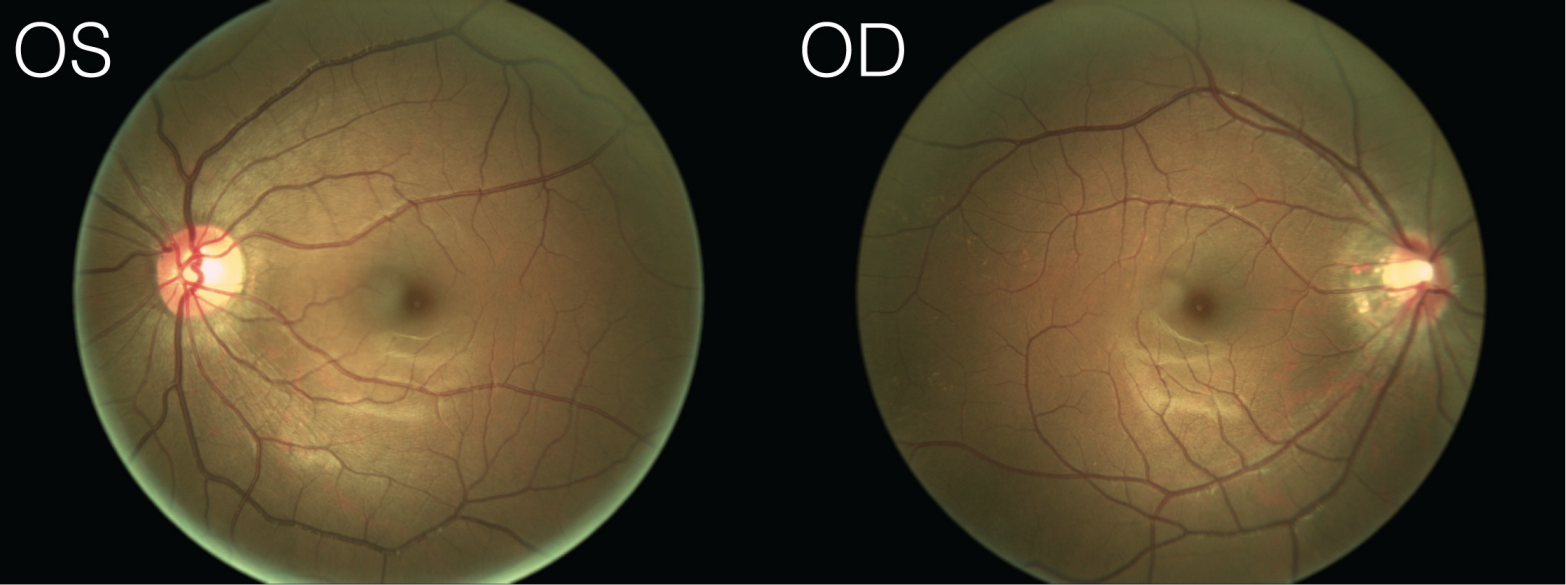
Raw fundus photographs. Unedited fundus photographs (OS = left eye, OD = right eye) obtained for our naïve observer.

The fundus photographs for our naïve observer (Fig 2C and Fig 5) were obtained using a NIDEK Microperimetry device (MP1). For visualization purposes, the retinal vasculature (Fig 2C overlay) was manually extracted from the photograph with Adobe Photoshop using a combination of image feature selection techniques, and the contrast and color balance of the photos was adjusted for visualization purposes. A raw version of the fundus photos is provided in Fig 5.

### Supplemental Experiment

**Fig 6:**
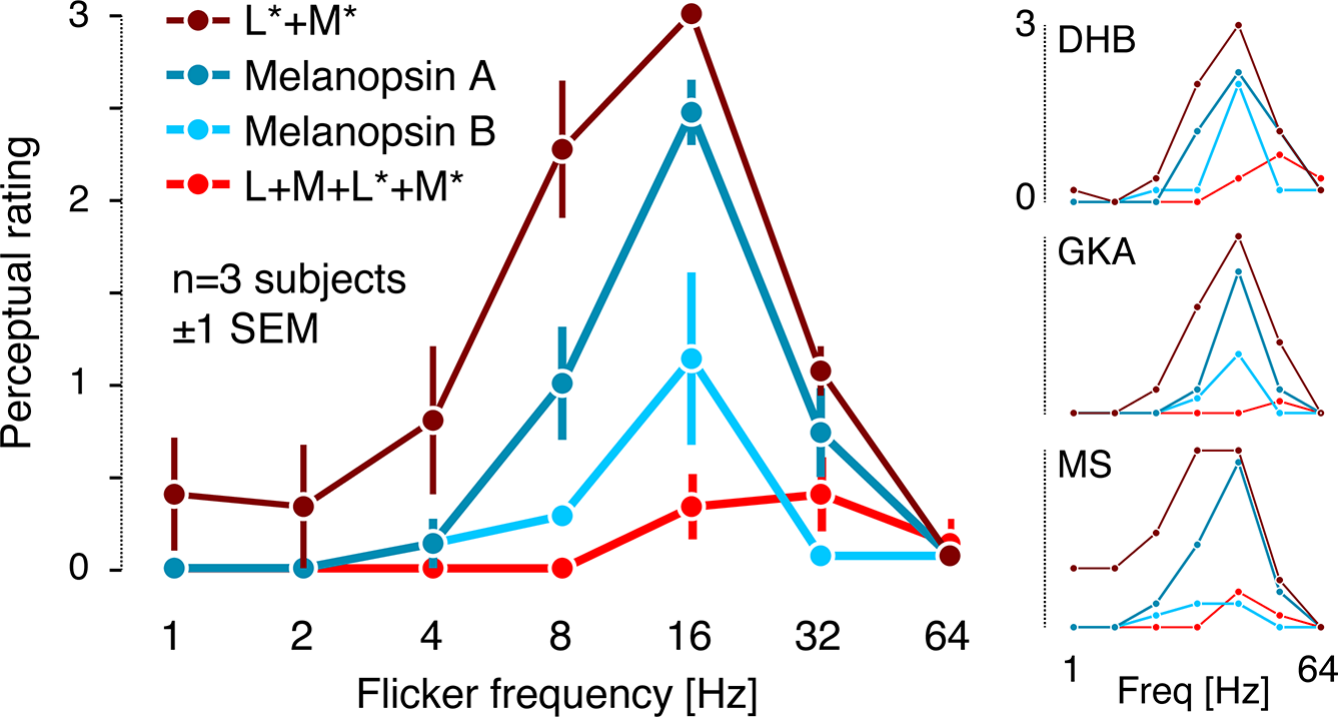
Rating data from the supplemental experiment. Average ratings across the three observers. L*+M*, penumbral L and M cone modulation; Melanopsin A, melanopsin-directed modulation that did not silence penumbral cones; Melanopsin B, melanopsin-directed modulation with penumbral cones silenced; L+M+L*+M*, modulation visible to both open-field and penumbral L and M cones. Individual observer ratings are shown to the right.

In a supplemental experiment, we tested whether a melanopsin-directed modulation designed to silence open-field but not penumbral cones can elicit percepts of the Purkinje tree, and whether this is due to contrast seen by penumbral cones (Fig 6). Observers viewed two melanopsin-directed modulations. The *Melanopsin A* modulation was constructed using a target contrast for melanopsin of 20% with open-field L, M and S cones silenced. Stimulus contrast was not constrained for penumbral cones, however, and this resulted in a *Melanopsin A* modulation that produced ~2–3% contrast on penumbral L and M cones and ~10% contrast on penumbral S cones (Table 1). The *Melanopsin B* modulation was constructed again using a target contrast of 20% for melanopsin, but with both open-field and penumbral cones silenced. As control modulations, we added the L and M penumbral cone directed modulation and a modulation stimulating both sets of L and M cones together while silencing the other photopigments. These latter two modulations were as in our main experiment.

The rating methods, background light level, and stimulus temporal properties were the same as in our main experiment, and the three authors again served as observers. Each combination of modulation direction and frequency was presented five times, with trial order randomized. On 14 out of 154 trials, no modulation at all was shown (blank trials).

We replicated the visibility of the Purkinje tree for selective penumbral L and M cone stimulation (Figs 3 and 6), finding a maximum visibility rating at 16 Hz, dropping sharply at frequencies lower than 8 Hz and higher than 32 Hz. As in the main experiment, our open-field and penumbral L and M cone modulation elicits some spatial structure at frequencies higher than 16 Hz, but no Purkinje tree. Crucially, the *Melanopsin A* modulation elicited Purkinje-tree percepts similar to those produced by the penumbral L and M cone modulation. When penumbral cones were silenced but melanopsin driven at the same contrast (*Melanopsin B*), the Purkinje-tree percept was considerably reduced, with some observer variability in the degree of reduction. We speculate that the individual variability results from individual differences in the residual penumbral cone contrast produced by the *Melanopsin B* modulation. Out of the 14 blank trials, observers MS and GKA rated all of them as 0; observer DHB rated one trial out of the blank trials as 1, and 13 as 0.
